# Dexterous manual movement facilitates information processing in the primary somatosensory cortex: A magnetoencephalographic study

**DOI:** 10.1111/ejn.15310

**Published:** 2021-06-04

**Authors:** Toshiaki Wasaka, Tetsuo Kida, Ryusuke Kakigi

**Affiliations:** ^1^ Department of Engineering Nagoya Institute of Technology Nagoya Japan; ^2^ Department of Integrative Physiology National Institute for Physiological Sciences Okazaki Japan; ^3^ Higher Brain Function Unit, Department of Functioning and Disability, Institute for Developmental Research Aichi Developmental Disability Center Kasugai Japan; ^4^ Section of Brain Function Information, Supportive Center for Brain Research National Institute for Physiological Sciences Okazaki Japan

**Keywords:** magnetoencephalography, manual dexterity, sensorimotor integration, tactile

## Abstract

The interaction between the somatosensory and motor systems is important for control of movement in humans. Cortical activity related to somatosensory response and sensory perception is modulated by the influence of movement executing mechanisms. This phenomenon has been observed as inhibition in the short‐latency components of somatosensory evoked potentials and magnetic fields (SEPs/SEFs). Although finger is the most dexterous among all the body parts, the sensorimotor integration underlying this dexterity has not yet been elucidated. The purpose of this study was to examine the sensorimotor integration mechanisms in the primary somatosensory cortex (SI) during simple and complicated finger movement. The participant performed tasks that involved picking up a wooden block (PM task) and picking up and turning the wooden block 180° (PTM task) using the right‐hand fingers. During these tasks, the SEFs following right median nerve stimulation were recorded using magnetoencephalography. The amplitude of the M20 and M30 components showed a significant reduction during both manual tasks compared to the stationary task, whereas the M38 component showed a significant enhancement in amplitude. Furthermore, the SEFs recorded during continuous rotation of the block (rotation task) revealed a characteristic pattern of SI activity that was first suppressed and then facilitated. Since this facilitation is noticeable during complicated movement of the fingers, this phenomenon is thought to underlie a neural mechanism related to finger dexterity.

## Graphical abstract

Although it is generally accepted that the effect on the short‐latency somatosensory evoked potentials and magnetic fields components with movement execution is inhibitory in nature, we found inhibition followed by facilitation in the primary somatosensory cortex during complex movements using the fingertips. The relationship between the somatosensory information processing and motor diversity of the fingers is the basis for fine coordinated movements, and our results offer insight into the elucidation of neural mechanisms underlying dexterous manual movement.

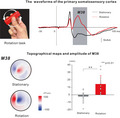

**Abbreviations**
ANOVA: analysis of variance
ECD: equivalent current dipole
MEG: magnetoencephalography
MI: primary motor cortex
MRI: magnetic resonance imaging
PM task: picking up and moving task
PTM: task picking up, turning, and moving task
SEPs: somatosensory evoked potentials
SEFs: somatosensory evoked magnetic fields
SI: primary somatosensory cortex

## 1 INTRODUCTION

Sensory information is important to the execution of movement. Proprioceptive and cutaneous information provided from the moving body parts is known to be modulated by pathways from the periphery to the somatosensory areas. While multiple studies have shown that the short‐latency components of somatosensory evoked potentials (SEPs) and somatosensory evoked magnetic fields (SEFs) decrease in amplitude prior to and during voluntary limb movement (Bocker et al., 1993; Kristeva‐Feige et al., 1996; Rushton et al., 1981; Tapia et al., 1987; Wasaka et al., 2003), a psychophysical study reported that voluntary movement reduced the detection threshold to tactile information from body parts (Angel & Malenka, 1982; Chapman et al., 1987). Such inhibition reportedly occurred in the primary somatosensory cortex (SI) when a stimulus is delivered to the nerve that is innervating the moving body part (Kakigi et al., 1997). This phenomenon is known as “gating.”

Motor output alters sensory information processing. Commands from the motor areas cause muscle contraction via the spinal cord, but at the same time have the function of adjusting the activity of the somatosensory system. This centrifugal mechanism leads to the assumption that the functional role of this inhibitory phenomenon is to regulate the inflow of somatosensory information from moving body parts so as not to induce an unintentional reflex via somatosensory feedback. In many previous studies, suppression of SEP/SEF components with voluntary movement used simple movements such as hand gripping (Wasaka et al., 2012) and finger extension/flexion (Cheron & Borenstein, 1987; Kakigi et al., 1995; Seyal et al., 1987), or plantar flexion/dorsiflexion of the lower limb (Wasaka et al., 2005). However, because the previous studies showed that cortical activation in sensorimotor areas during simple finger movement is different from that during complex finger movements (Shibasaki et al., 1993), the neural mechanisms involved in the sensorimotor interaction of skilled complex movement may differ from that of simple movement. It remains to be clarified how sensorimotor integration is used to perform skillful movement.

The finger is one of the most dexterous body parts in humans, and manual movement in daily life is coordinated by independent finger movements, so we hypothesized that the neural background of skillful manual movement involved in the specific sensorimotor integration differs from that of other body parts. In a finding suggesting that the modification of somatosensory information processing with movement may be more complicated than conventional simple suppression, our previous study found that the initial components, M20 and M30, decreased and a subsequent component approximately 40 ms increased when manipulating an object with the hand, e.g., with the palm and fingers (Wasaka et al., 2017). This phenomenon was not observed when simply grasping an object or moving the fingers in a complicated manner. From these results, we presumed that the increase in activity in SI involved manipulating an object with the fingertips. However, the exact cause of facilitation in SI activity remains unknown. Elucidating this issue is important for clarifying the neural background of skilled movements. The purpose of this study is to clarify whether the difference in complexity of fingertip movements affects SI activation by comparing brain activity during a relatively simple manual task of picking and releasing an object and during the complicated manipulation of an object (turning an object) using the fingertips.

The characteristics of finger movement are prehension, grasping, and manipulating objects. In this experiment, we focused on simple and complex manual manipulative movements using the thumb, index, and middle fingers. In the first experiment, we focused on the types of finger movements used when manipulating an object, aiming to clarify what factor of movement changed the SI activities. To investigate the activation in the somatosensory areas, we stimulated the median nerve that innervates those three fingers. In the second experiment, we used a continuous rotation task based on the hypothesis that complex manual movements facilitate SI activation. We investigated the changes in somatosensory information processing when performing only complex manual movement, and showed the characteristics of sensorimotor integration by comparing the temporal activation pattern in the SI between stationary and dexterous finger movements using the fingertips.

## 2 METHODS

### 2.1 Participants

Both experiments were performed with 13 healthy right‐handed volunteers (five females and eight males, mean age 28.8 ± 8.9 years). Each volunteer was in good health and free of medication before the experiment. We obtained written informed consent from all the volunteers for participation in the experiment, which was approved by the Ethics Committee of the Nagoya Institute of Technology (26‐005).

### 2.2 Stimulation

The right median nerve was electrically stimulated on the palmer aspect of the wrist with a saddle type electrode (NM‐422B, Nihon Koden). The cathode was placed 2 cm proximal to the anode. With constant current square wave pulses (duration, 0.2 ms) being provided, with an interstimulus interval being 500 ms, the electrode was fixed on the wrist during the recording. The stimulus intensity was adjusted to the motor threshold which produced a slight twitch of the abductor pollicis brevis muscle. The volunteers were asked to concentrate on the manual movement and not to pay attention to the continuous electrical stimulation.

### 2.3 Experimental design

Experiments were conducted in a quiet, magnetically shielded room. The participants performed two experiments. Both experiments were carried out on the same day.

#### 2.3.1 Experiment 1

SEFs were recorded in each of the following conditions. In the picking up and moving task (PM task), the participant was asked to repeatedly pick up the middle of a wooden block (cuboid, 2 × 2 × 7 cm in size, weight 18 g) using their right fingers, move, and release it. When moving and releasing the block, the participant was instructed to insert it into a dent vertically and prevent it from falling. The distance between the two dents was 3 cm and the dents were located in front‐back direction. During the PM task, they continuously moved a wooden block between the two dents. In the picking up, turning, and moving task (PTM task), the participant repeatedly picked up the middle of a wooden block, rotated it 180° using the picking up position as a fulcrum, moved it 3 cm, and released it. The PTM task added rotation of the wooden block in the middle of the PM task. Both PM and PTM tasks were performed using only the thumb, index, and middle finger of the right hand, while the left hand was resting on the table. Participants were instructed to perform both the tasks at a speed that could be performed accurately without dropping the block. We also recorded SEFs during the stationary state (stationary task) with both hands on the table. The order of the motor and stationary tasks was randomized among the participants. The duration of each task was approximately 3 min.

#### 2.3.2 Experiment 2

The participants performed two tasks. In the rotation task, they continuously rotated a wooden block (cuboid, 2 × 2 × 7 cm in size, weight 18 g) using the right thumb, index, and middle fingers. Participants were asked to pick the middle of the block and rotate it using this position as a fulcrum at their own pace, but maintaining a constant speed. In the stationary task, the participant was instructed to keep both hands stationary on the table. The duration of each task was approximately 3 min. The two tasks were performed randomly by the participants.

### 2.4 MEG acquisition

Neural activity was measured using a helmet‐shaped 306‐channel magnetoencephalography (MEG) system (Vectorview, Elekta Neuromag Yo), which was comprised of 102 identical triple sensor elements. A sensor element in each recording position consisted of one magnetometer and two orthogonal planar‐type gradiometers (one for latitudinal and the other for longitudinal directions of changes in neuromagnetic signals). These planar gradiometers are sufficiently powerful to detect the largest signal just over local cerebral sources. The continuous data were recorded with a bandpass filter of 0.03–300 Hz and digitized at 1,000 Hz. Large noise included in the recorded signal was removed using the signal space projection technique. Magnetic resonance images (MRIs) were obtained with a 3‐tesla MRI system (Allegra; Siemens). Prior to the acquisition of MEG data, the co‐registration procedure of MEG sensor, head, and MRI coordinate systems was performed.

### 2.5 MEG analysis

We analyzed the MEG signals recorded from 204 planar‐type gradiometers. Trials in which the signal variations were larger than 3,000 fT/cm were excluded from the averaging. The analysis time was 300 ms, which included a prestimulus period of 100 ms. The data from the 100 ms before the onset of stimuli were used to calculate the baseline. A total of 300 artifact‐free epochs were averaged to obtain SEF waveforms.

In the source‐space analysis, to identify the equivalent current dipoles (ECDs) in the MEG components, the sources of measured responses were modeled using the time‐varying current dipoles method (Hamalainen et al., 1993). The earliest deflection of MEG waveforms was identified at around 20 ms in all participants. The ECDs that best explained the most dominant signals were determined by a least‐squares search, based on 16–18 channels around the channel. Only ECDs which accounted for more than 80% of the goodness‐of‐fitness in a channel subset were accepted. Then, all MEG channels were used to compute the time‐varying dipole model allowing the strengths of the previously found ECDs to change over the entire period of analysis, while the source location and orientations were kept fixed.

The moment of ECDs was measured for prominent peaks. Three components were identified in all participants. The first deflection peaked at approximately 20 ms (M20). The second‐largest component was at approximately 30 ms (M30). The subsequent component was a small deflection peaking approximately 38 ms (M38). In experiment 1, to compare the peak amplitude of the three components among the stationary and two motor tasks, we used a one‐way repeated measures analysis of variance (ANOVA), with “Task” (PM, PTM, or stationary task) as the factor. To analyze the assumption of sphericity prior to the repeated ANOVA, we used Mauchly's test of sphericity. For multiple post hoc comparisons, the Bonferroni test was used. In experiment 2, in order to compare the source waveforms in the SI between the stationary and rotation tasks, we used the paired *t* test at each time point (every 1 ms). We also compared the peak amplitude of the three components of the two tasks using paired *t* tests. The level of statistical significance was set at 5% (*p* < 0.05).

## 3 RESULTS

### 3.1 Experiment 1

**FIGURE 1:**
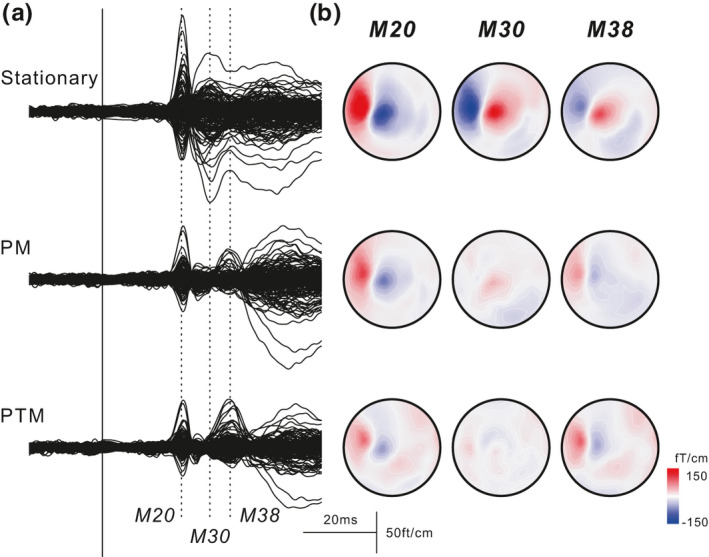
Superimposed grand‐averaged somatosensory evoked magnetic field waveforms (13 participants) in the stationary task and during two manual tasks (a) and topographical maps of M20, M30, and M38 components (b) in Experiment 1. The two tasks were picking up and moving a wooden block (PM task) and repeatedly picking up, turning, and moving a wooden (PTM task). The first deflection, peaking at around 20 ms (M20) following stimulation, was observed. While the subsequent component (M30) was clearly identified under the stationary task, those in the other tasks were decreased. In contrast, the component that peaked around 38 ms (M38) appeared in the two manual tasks. The topographical map of M38 shows pattern reversal between the stationary condition and the two tasks

The superimposed SEF waveforms and the topographical maps are shown in Figure 1. In the stationary task, the first deflection (M20) and the subsequent deflection (M30) were identified. These components showed a decrease under the PM and PTM tasks, while a large deflection appeared at approximately 38 ms (M38). The topographic map showed a clear influx and outflux pattern in three components. The topographical patterns of M20 were the same in all three tasks, whereas those of M30 and M38 showed completely different patterns. It is noteworthy that M38 showed an influx and outflux pattern reversal between the stationary and PTM tasks.

**FIGURE 2:**
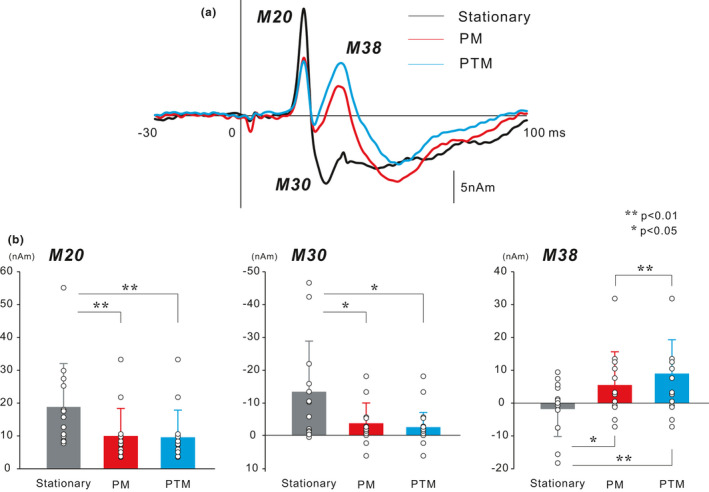
(a) The temporal change of the grand‐averaged equivalent current dipole (ECD) waveforms of the primary somatosensory cortex (SI) in Experiment 1 (13 participants). While the peak amplitude of M30 in the two manual tasks decreased compared to the stationary task, the peak amplitude of M38 increased with manual movement. (b) Mean amplitude of the ECD components of the SI. Vertical lines indicate standard deviations. Open circles represent individual data. Statistical comparison showed that the peak amplitude of the ECD moments for M20 and M30 in the PM and PTM tasks was significant smaller than that in the stationary task. In contrast, the peak amplitude of the ECD moment for M38 showed a significant enhancement in the two manual tasks. The results are available in Figshare, along with the data

The ECD for the first deflection was estimated around the posterior bank of the central sulcus. Figure 2 shows the grand‐averaged ECD waveforms of the SI in the stationary, PM, and PTM tasks. First, we conducted a one‐way repeated measures ANOVA to compare the modulation of each component in the two tasks with the stationary task. The results showed a significant main effect for the peak ECD moment for M20 (*F*_(2,24)_ = 22.370, *p* < 0.01, *ε* = 0.612), M30 (*F*_(2,24)_ = 10.145, *p* < 0.01, *ε* = 0.523), and M38 (*F*_(2,24)_ = 17.636, *p* < 0.01, *ε* = 0.596). Bonferroni comparison revealed that the peak of ECD moment of M20 was significantly smaller in the PM (*p* < 0.01) and PTM tasks (*p* < 0.01) than in the stationary task, and that of M30 was significantly smaller in the PM (*p* < 0.05) and PTM (*p* < 0.05) tasks than in the stationary task. For both components, no difference was observed between the PM and PTM tasks. In contrast, the peak of ECD moment of M38 was significantly enhanced in the PM (*p* < 0.05) and PTM tasks (*p* < 0.01) compared with the stationary task, and the PTM task showed a significantly higher amplitude than the PM task (*p* < 0.01).

### 3.2 Experiment 2

**FIGURE 3:**
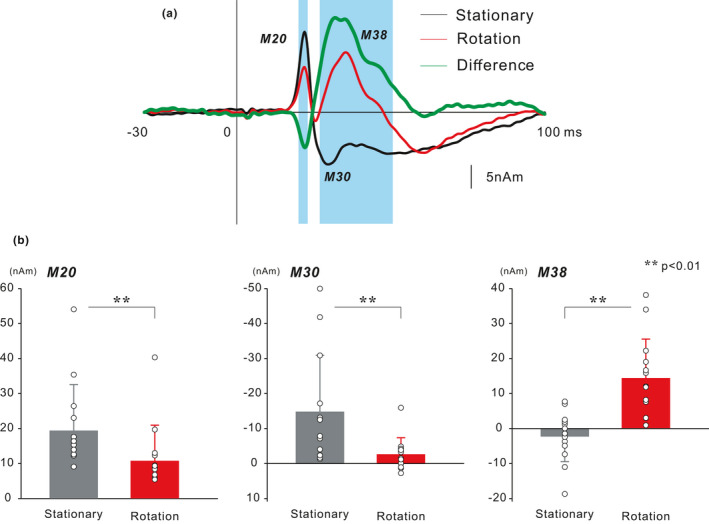
(a) The temporal change of the grand‐averaged ECD waveforms of the SI (13 participants) in the stationary and rotation tasks and the subtracted waveform in Experiment 2. Paired *t* test between the two tasks at each point showed that the latencies of the deflections were nominally significant at the level of *p* < 0.05 (shaded areas). (b) Mean amplitude of the ECD components of the SI in the stationary and rotation tasks. Vertical lines indicate standard deviations. Open circles represent individual data. Statistical comparisons showed that the peak amplitudes of the ECD moments for M20 and M30 in the rotation task was significant smaller than that in the stationary task. In contrast, the peak amplitude of the ECD moment for M38 was significantly enhanced in the two manual tasks. The results, along with the data, are available in Figshare. ECD, equivalent current dipole; SI, primary somatosensory cortex

The grand‐averaged ECD waveforms in the rotation and stationary tasks are shown in Figure 3, and we computed the different waveforms induced by the subtraction of stationary waveforms from rotation waveforms. The comparison of peak amplitude revealed that M20 and M30 were significantly smaller in the rotation task than in the stationary task (*p* < 0.01), and M38 showed a significant enhancement in the rotation task (*p* < 0.01). These results are in line with those of Experiment 1. To compare time series activity in the SI between the stationary and rotation tasks, we used a paired *t* test. The results showed a significant difference between 20 to 23 ms and 27 to 51 ms. The difference waveform exhibited a decrease followed by an increase in the SI activity.

## 4 DISCUSSION

The inflow of somatosensory information during voluntary movement is modulated in the central nervous system, including the cuneate nucleus in cats (Ghez & Pisa, 1972) and the spinal cord (Seki & Fetz, 2003) and somatosensory cortex in monkeys (Chapman et al., 1988; Jiang et al., 1991). It is generally accepted that the effect on the short‐latency SEPs/SEFs components with movement execution is inhibitory in nature, and the functional role of this modulation is considered to filter the sensory input that is not related to movement execution in order to perform fine movement. In humans, short‐latency components of SEPs/SEFs are suppressed during voluntary movement. However, little evidence exists regarding the sensorimotor integration of dexterous finger movement. The relationship between the somatosensory information processing and motor diversity of the fingers is the basis for fine coordinated movements, it is possible that sensorimotor integration, i.e., the combination of inhibition and facilitation in the SI, involved in the neural background of the motor diversity of the fingers.

The present study investigated whether manipulating an object with the fingers causes a specific phenomenon that is different from the inhibition of the SI that has been previously reported. Our results revealed that the SEF component of M38 was significantly enhanced during dexterous finger movement, while the preceding components showed significant reductions. This means that the activity of SI is not only suppressed but also increased when manipulating an object by dexterous finger movement.

### 4.1 Attenuation of M20 and M30

While the change in amplitude of the primary cortical component in SI with muscle contraction has been controversial, the central nervous system has a neural mechanism that modulates the inflow of somatosensory information from moving body parts in order to suppress unnecessary reflex and muscle contraction induced by reafferent signals. Some studies reported that the SEPs/SEFs component around 20 ms did not change (Cheron & Borenstein, 1987, 1991; Cohen & Starr, 1987; Kirimoto et al., 2014; Sugawara et al., 2016; Takahara et al., 2020; Tapia et al., 1987), while others reported an attenuation in the amplitude (Abbruzzese et al., 1981; Hoshiyama & Kakigi, 1999; Huttunen & Homberg, 1991; Jones, 1981; Lei et al., 2018; Seyal et al., 1987). Both results were also included in our previous studies using many kinds of manual movements (Wasaka et al., 2017). Significant inhibition was found only in the ball rotation task compared with the stationary task, whereas other motor tasks, such as ball grasping and simple finger flexion/extension, showed no changes in amplitude. In the ball rotation task, the fingertips touched the balls to manipulate them, but under the ball gripping task, the fingertips did not touch the balls because they were small. With gain reduction of this SEPs/SEFs component being seen during dexterous manual movements, such as exploring and manipulating objects using the fingertips, the results of the present study also indicated a significant reduction of M20 in the PM and PTM tasks. These facts may help to explain that somatosensory input from the fingertips modulates activity in relation to M20. The first cortical stage of information processing occurs at around 20 ms in area 3b (Allison et al., 1991). This area receives information from receptors in the skin (Iwamura et al., 1993), which are densely populated at the fingertips. Taken together, the large amount of somatosensory information generated from the fingertips may be responsible for the decrease of M20 during finger movement.

Attenuation of the subsequent component at approximately 30 ms with muscle contraction has consistently been reported (Cheron & Borenstein, 1987; Hoshiyama & Sheean, 1998; Seyal et al., 1987; Sugawara et al., 2016). Our results also showed a decrease in M30. This component is known to be more sensitive than M20 to effects of movement. The current source for M30 has not yet been clarified, however, previous studies have reported that area 4 of the primary motor cortex (MI) (Kawamura et al., 1996) or area 3b in the postcentral sensory cortex (Kakigi, 1994) may be involved in generating M30. Owing to the fact that the amplitudes of M20 and M30 show distinctive behaviors in response to changes in stimulus intensity levels (Lin et al., 2003) and interstimulus intervals (Wikstrom et al., 1996), the difference in modulation during voluntary movement may be caused by a difference in neural sources.

The gain reduction of SEPs/SEFs with voluntary movement was dependent on the parameters of muscle contraction. Large amounts of contractile force (Cohen & Starr, 1985; Sakamoto et al., 2004; Tinazzi et al., 1998; Wasaka et al., 2005) or contraction velocity (Angel & Malenka, 1982; Rauch et al., 1985; Staines, Brooke, Cheng, et al., 1997; Staines, Brooke, Misiaszek, et al., 1997) have been shown to strongly inhibit of SEP/SEF components due to a centrifugal mechanism. In the PTM task, the action of turning the wooded block was added to the PM task, and the complexity of finger movement was relatively high. However, the results showed no significant difference in M20 and M30 amplitude between the two tasks. It is assumed that the gain reduction of these components is saturated with finger pinching and the effect of more complicated finger movement may not be strongly reflected.

### 4.2 Enhancement of M38

The modulation of SEP/SEF amplitude with movement could occur in two possible ways: (a) occlusion between the induced somatosensory afferents following electrical stimulation and the afferent signals from the muscles, joints, and skin induced by movement (centripetal gating); and (b) interaction between the given sensory signals and the efferent signals induced by the motor command from the motor‐related areas (centrifugal gating) (Jones et al., 1989). Because SEFs were recorded during movement, the amplitude should be reduced by the centripetal gating effect, whereby afferent information from cutaneous and muscle spindle generated by voluntary movement modulates neural activity, which generates the evoked response following electrical stimulation. However, M38 in the PM and PTM tasks was significantly enhanced. Other mechanisms may be involved in this facilitatory modulation.

One possibility for the increase is the attentional effect. Typically, selective attention toward somatosensory input produces an increase in the amplitude of evoked potential, not a reduction (Desmedt & Tomberg, 1989; Garcia‐Larrea et al., 1991; Schubert et al., 2008; Zopf et al., 2004). Prior to the experiment, participants were instructed to concentrate on the manual tasks and to not pay attention to the electrical stimulation, and after the experiment, they reported that they were able to do so. It is unlikely that attention to electrical stimulation increased the M38. In a finding that could be explained by the fact that the amplitude of the short‐latency SEP component has been shown to be facilitated as well as inhibited depending on the kinesthetic requirements to control movement execution (Legon & Staines, 2006; Staines, Brooke, Cheng, et al., 1997; Staines, Brooke, Misiaszek, et al., 1997), another possibility is that centrifugal mechanism modulates somatosensory information processing in relation to the demands on sensory inputs to perform motor tasks. It would likewise be conceivable that the demand for somatosensory information needed to control sensory‐guided behavior led to enhancement rather than suppression of the amplitude of the early SEF component.

The specificity of the fingers could be one possible explanation for facilitation of SI activity. We focused on movement using the fingertips, and the same fingers were used for both the PM and the PTM tasks, but the usage of finger movement is different. The thumb, index, and middle fingers moved simultaneously in the PM task, but these fingers moved independently when rotating the block in the PTM task. Owing to the long break between Experiment 1 and 2, we did not perform a statistical comparison among the motor tasks, but M38 amplitude showed the largest value in the rotation task of Experiment 2. The results revealed that M38 was larger when manipulating the object with complicated manual movements compared to when using simple pinching movements. In addition, there is evidence that facilitation of the SI component occurs during manual exploration of objects (Huttunen & Homberg, 1991; Knecht et al., 1993). Successful and efficient object manipulation requires precise modulation and temporal control of finger forces and movements, and there is the possibility that the M38 is enhanced because somatosensory information from these fingers was more necessary to dexterously move the fingers in the PTM task.

A previous study that used dipole modeling revealed that the anatomic generators of human SEPs/SEFs in the range of 20 to 40 ms involved a temporal relationship of activity in Brodmann's area 3b, 4, 1, and posterior parietal cortex (Inui et al., 2004). Using intracranial SEPs recording in humans, it has been reported that P25‐N35 is produced by a radially oriented generator located in the anterior crown of the postcentral gyrus in area 1 (Allison et al., 1989). Although the peak latency of our M38 was similar to that of N35, it is uncertain whether they are the same components because MEG cannot detect neural activities from radial dipoles such as those in the crown of the gyrus. In this study, although we tried to estimate the neural source of M38, we could not obtain a reliable model of temporal activation in multiple areas. Some studies have suggested that responses around 30 ms may be generated in the precentral motor cortex (Desmedt & Cheron, 1981; Dinner et al., 1987; Waberski et al., 1999). In addition, a functional MRI study has demonstrated the activation of SI as well as MI by median nerve stimulation (Spiegel et al., 1999). Although the neural mechanism and origin of M38 were not clearly elucidated, we assumed that the M38 has a different function from M20 and M30 because of the characteristic modulation with manual movement.

### 4.3 Temporal activation in the SI during skilled manual movement

Differences in activity caused by dexterous finger movement in SEFs were observed at 20–23 ms and at 27–51 ms in comparison with the rotation and stationary tasks. We assumed that the first time period reflects a reduction in M20, and that the second one was due to a reduction in M30 and an increase in M38. We revealed that sensorimotor integration in the SI was not a simple inhibition of the incoming somatosensory feedback, but a neural mechanism that facilitates the somatosensory information processing. The SI is cytoarchitecturally divided into areas 3, 1, and 2 and the somatosensory information is processed hierarchically. Although somatosensory cortical areas 3b and 1 represent the initial stages for tactile information processing, these areas also exhibit distinct structural and functional organization features. Digits are represented separately and independently from each other in area 3b, whereas there is a systematic increase in the complexity of the receptive field properties in area 1 (Ashaber et al., 2014; Iwamura et al., 1993). Since three fingers moved collaboratively in the rotation task, it is assumed that modulation was revealed at the stage where the interdigital integration occurred in the hierarchical processing of somatosensory information.

Afferent information from the muscle spindle and skin is an important feedback for controlling finger movement, and this information is responsible for the changes in M38 during manual dexterous movement. We stimulated the median nerve for the SEF recording, which conveys information from both cutaneous and proprioceptive receptors, and thus we could not confirm which somatosensory information induced the enhancement of M38. However, Confais et al. (2017) showed that facilitation of proprioceptive information processing in the SI occurs during voluntary movement, whereas cutaneous information processing results in inhibition. Where skillful and coordinated manual object manipulation is a special feature in humans, the increase in M38 may be due to a difference in the type of somatosensory information processing used for motor control.

Since it is difficult to perform dexterous movements using body parts other than the fingers, it remains to be seen whether this phenomenon is observed only during manipulative finger movement. However, since it is conceivable that complicated manual movement requires more somatosensory information than simple movement, we assumed that this facilitatory phenomenon plays an important role in performing dexterous movements. Since cortical excitability depends on the phase and type of movement (Behrendt et al., 2014; Seki et al., 2012), the activity of somatosensory areas is influenced by the motor system. However, we could not assess the relationship between SEF modulation and movement phase. Investigating how the sensorimotor integration depends on the movement phase will be important for elucidating the detailed neural mechanism of dexterous movement. Further studies will be needed to clarify the neural mechanism of sensorimotor integration underlying dexterous movement in humans.

## 5 CONCLUSION

The findings of our study reveal that information processing in the SI during manual movement is facilitated after inhibition. Additionally, when manipulating an object using the fingertips, the degree of facilitation increases based on the complexity of movement. Sensory information is crucial for motor control and learning, and our results offer insight into the elucidation of neural mechanisms underlying skillful movement, training for improvement of finger dexterity, and rehabilitation.

## CONFLICT OF INTEREST

The authors declare no competing financial interests.

## AUTHOR CONTRIBUTIONS

TW conceived and designed the experiments. TW and TK collected the data. TW analyzed the data. TW, TK, and RK interpreted the data. TW wrote the manuscript. All authors reviewed the manuscript.

### PEER REVIEW

The peer review history for this article is available at https://publons.com/publon/10.1111/ejn.15310.

## Data Availability

Data are accessible at Figshare: http://doi.org/10.6084/m9.figshare.14515722.
